# Supplementary material for: V-ATPase V0a1 promotes Weibel–Palade body biogenesis through the regulation of membrane fission
Source: eLife. 2021 Dec 14;10:e71526. doi: 10.7554/eLife.71526 (PMC8718113; doi:10.7554/eLife.71526)
Supplement: Figure 1—figure supplement 2—source data 1. [file elife-71526-fig1-figsupp2-data1.zip › Fig 1-fig supp 2_labeled.pptx]

## Slide 1
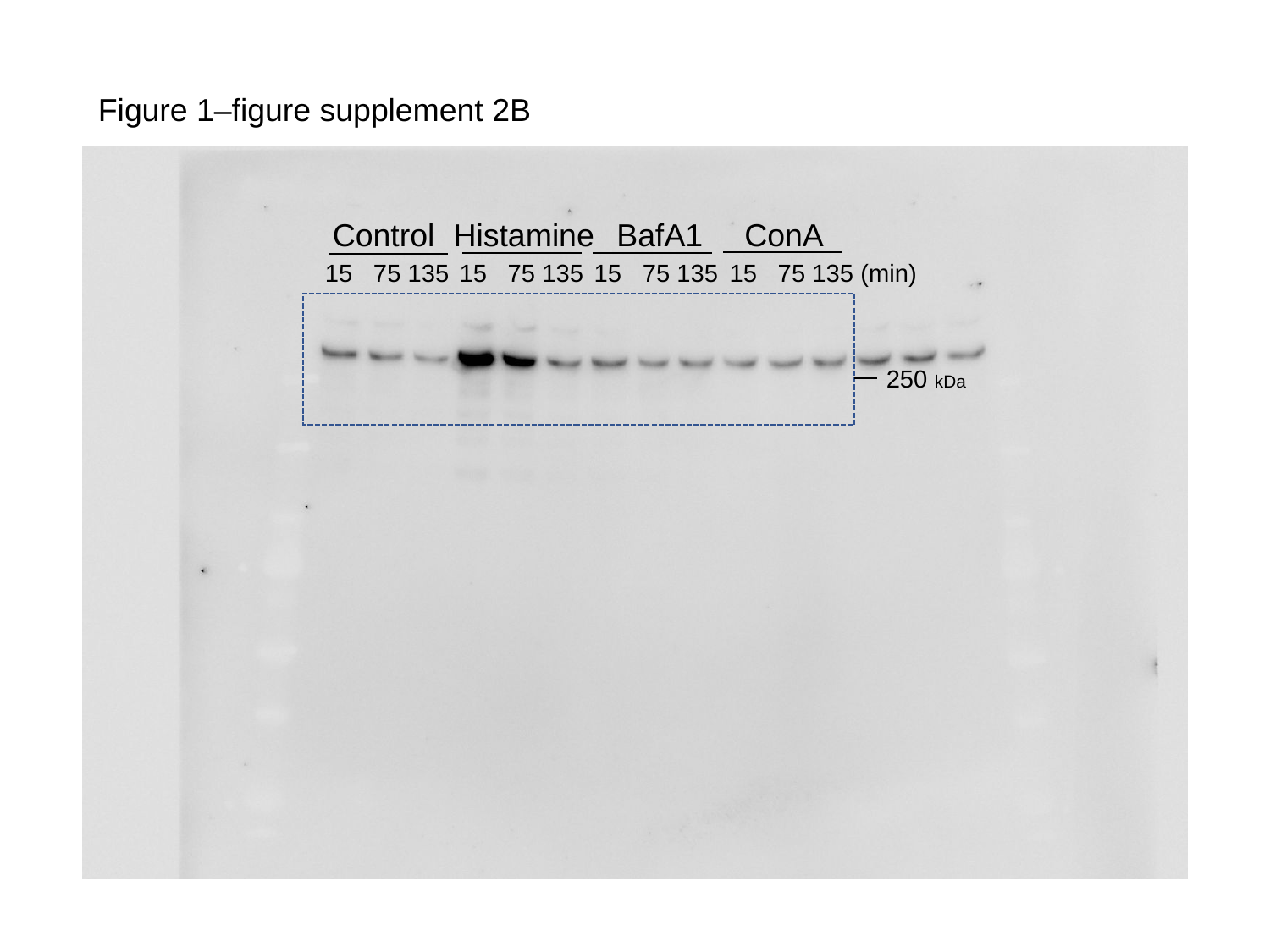

Figure 1–figure supplement 2B
Control
Histamine
BafA1
ConA
15 75 135
15 75 135
15 75 135
15 75 135 (min)
250 kDa

## Slide 2
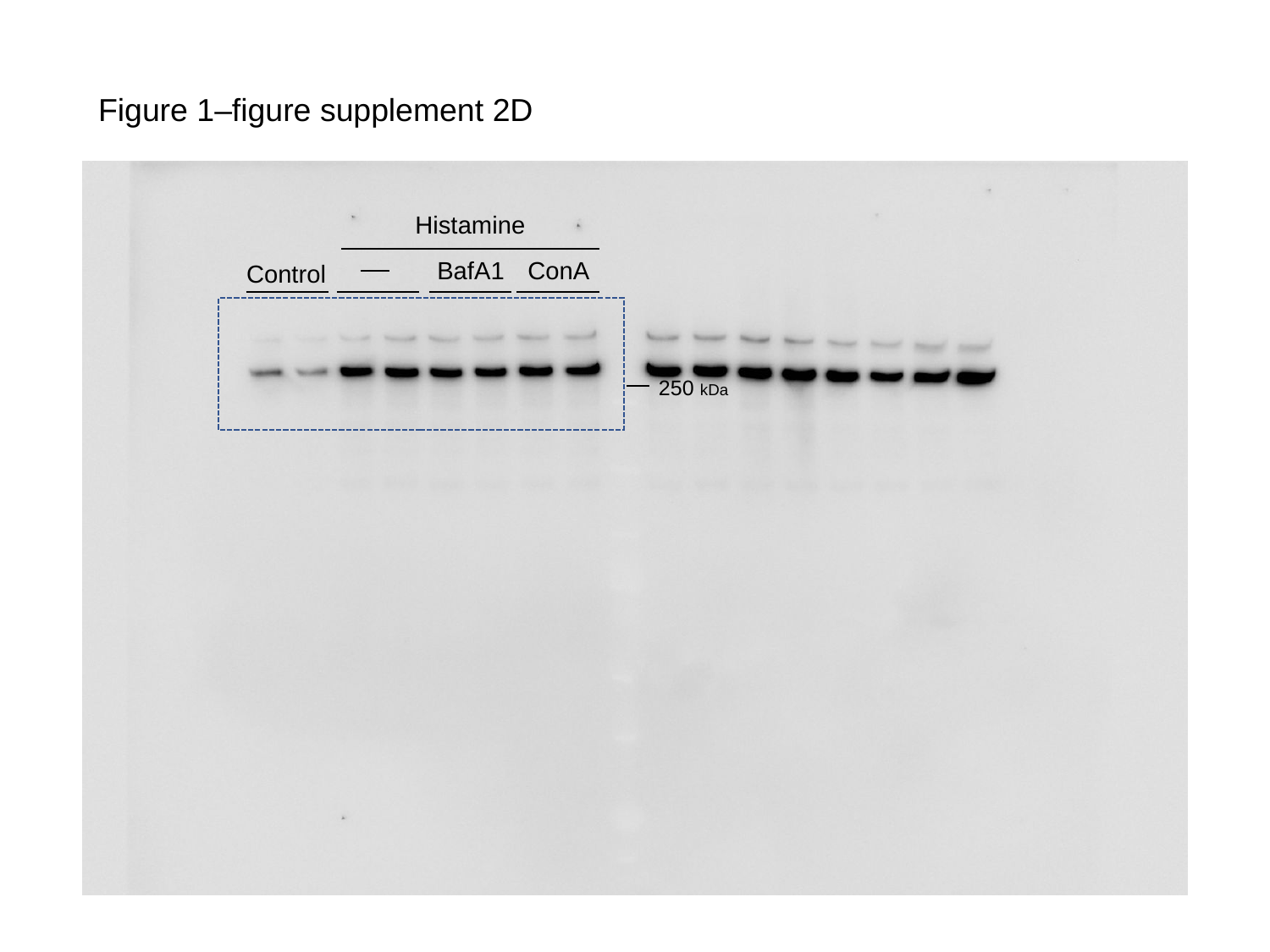

Figure 1–figure supplement 2D
Histamine
BafA1
ConA
Control
250 kDa
