# Supplementary figures and images for: V-ATPase V0a1 promotes Weibel–Palade body biogenesis through the regulation of membrane fission
Source: eLife. 2021 Dec 14;10:e71526. doi: 10.7554/eLife.71526 (PMC8718113; doi:10.7554/eLife.71526)

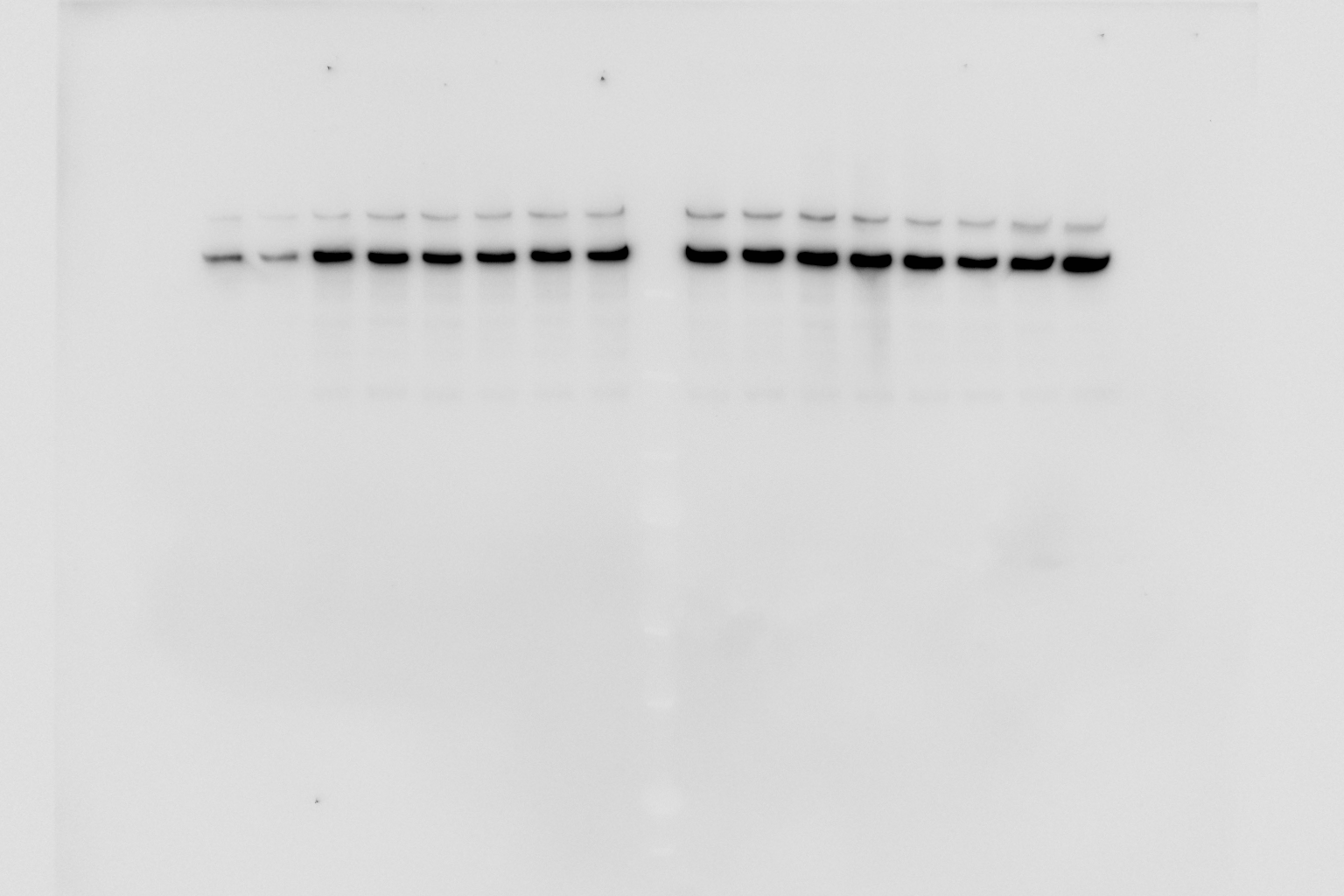

Supplement: Figure 1—figure supplement 2—source data 1. [file elife-71526-fig1-figsupp2-data1.zip › Fig1-fig supp 2D.tif]

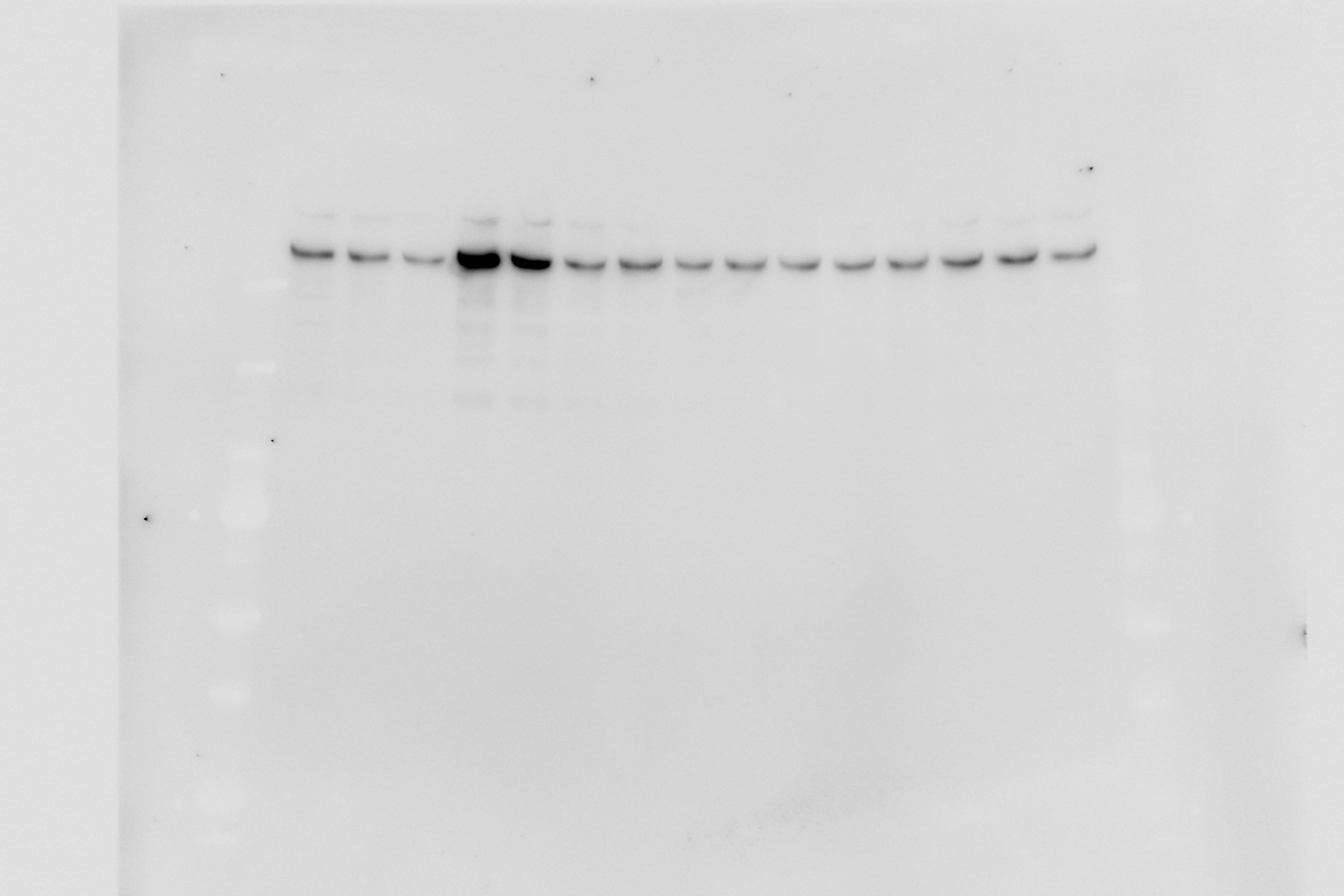

Supplement: Figure 1—figure supplement 2—source data 1. [file elife-71526-fig1-figsupp2-data1.zip › Fig1-fig supp 2B.tif]

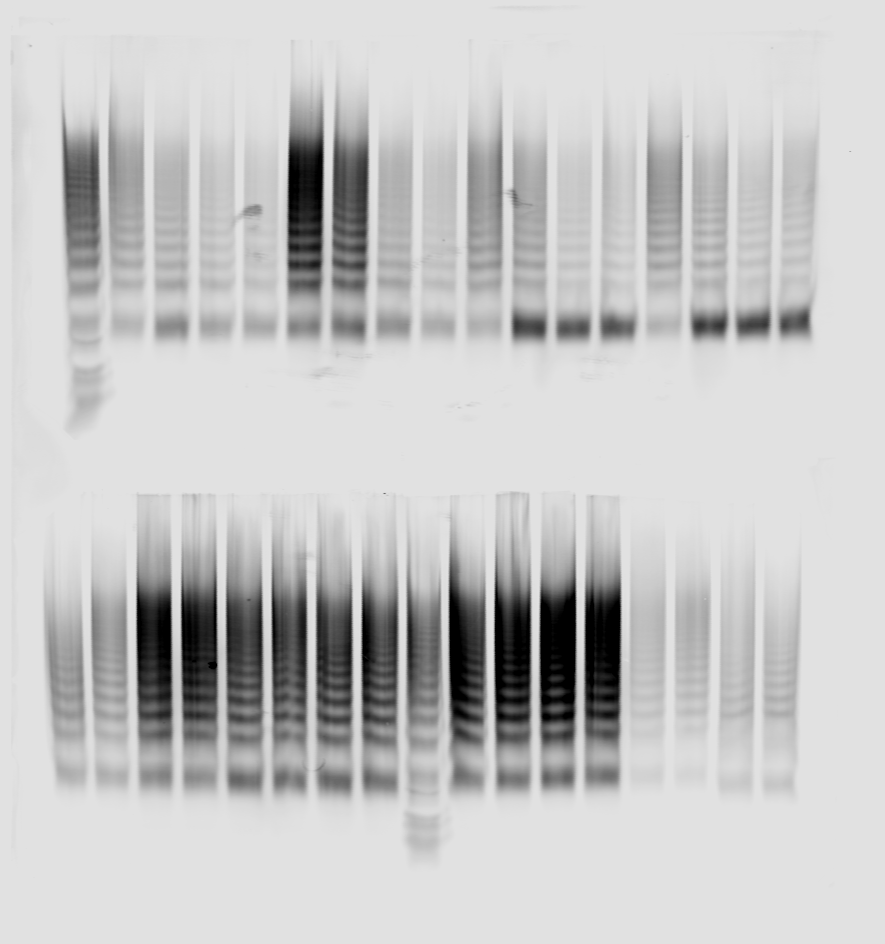

Supplement: Figure 2—figure supplement 1—source data 1. [file elife-71526-fig2-figsupp1-data1.zip › Fig2-fig supp 1A.tif]

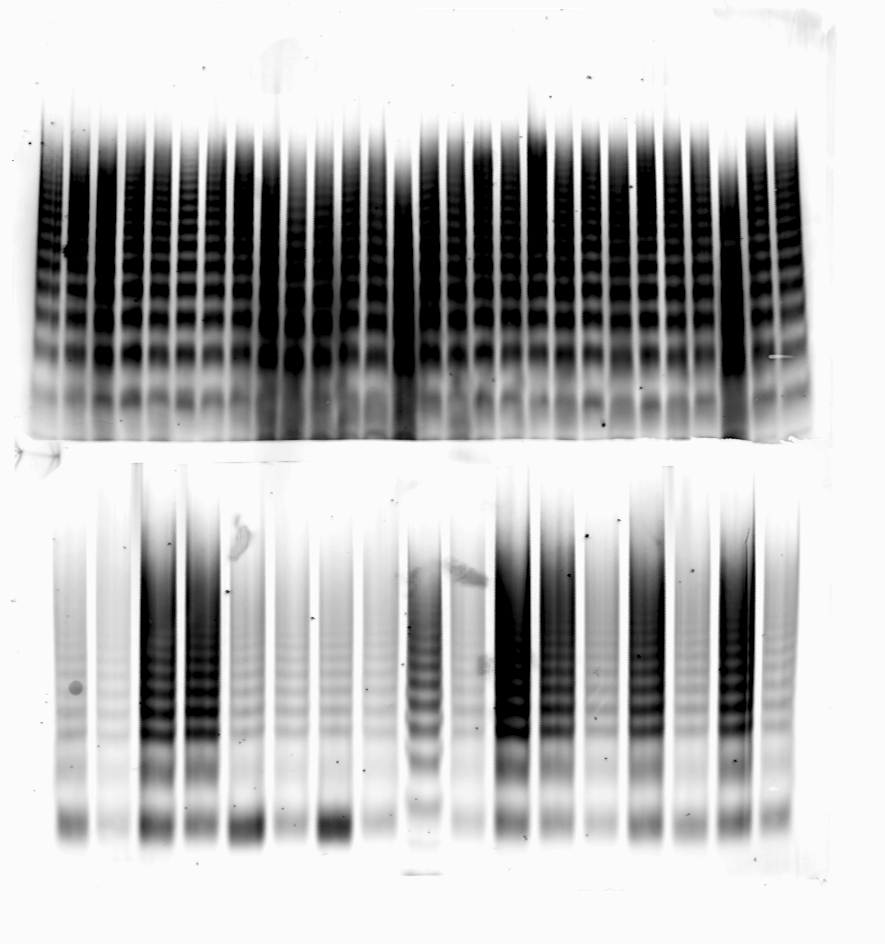

Supplement: Figure 2—figure supplement 1—source data 1. [file elife-71526-fig2-figsupp1-data1.zip › Fig2-fig supp 1C.tif]

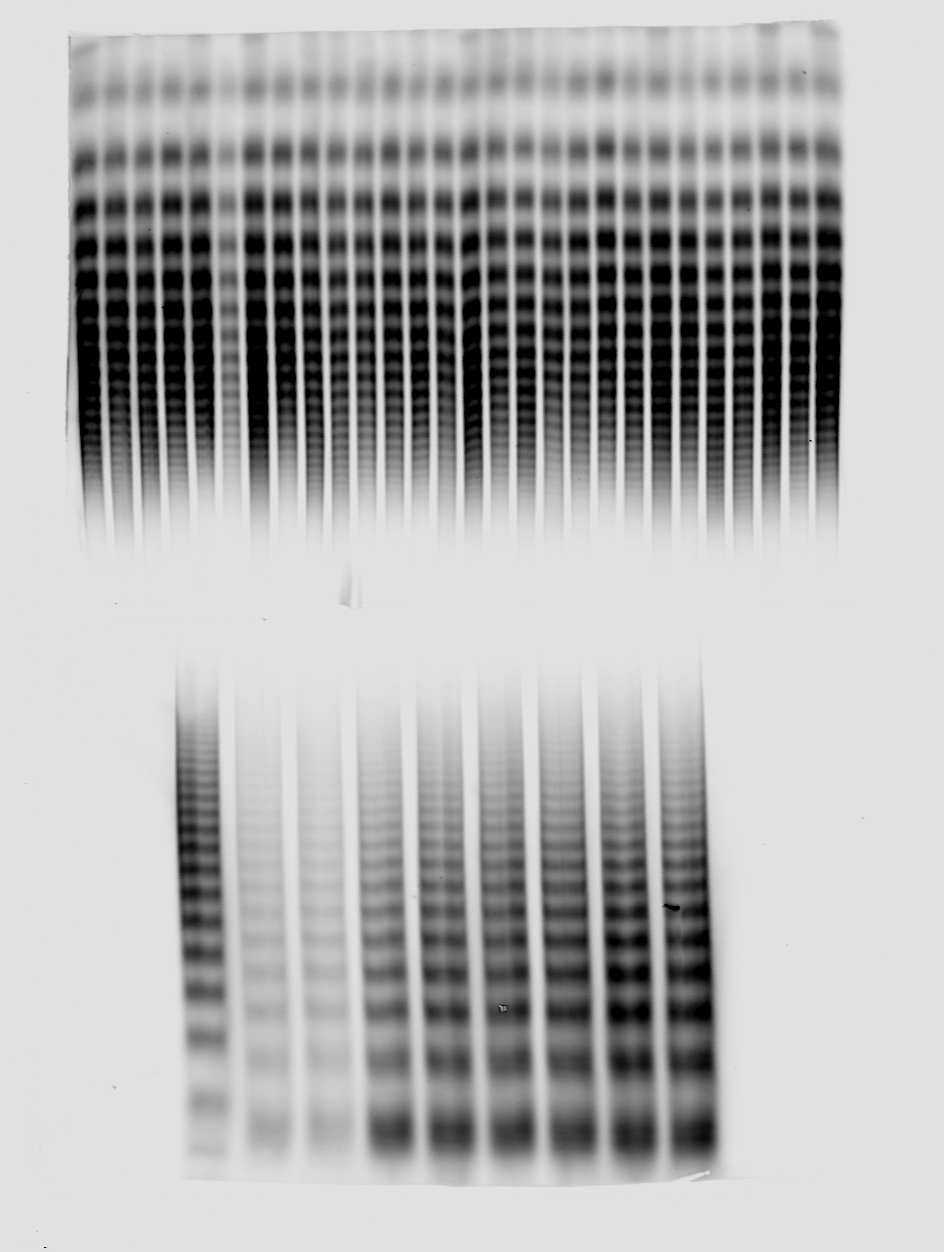

Supplement: Figure 3—figure supplement 1—source data 1. [file elife-71526-fig3-figsupp1-data1.zip › Fig3-fig supp 1B_left.tif]

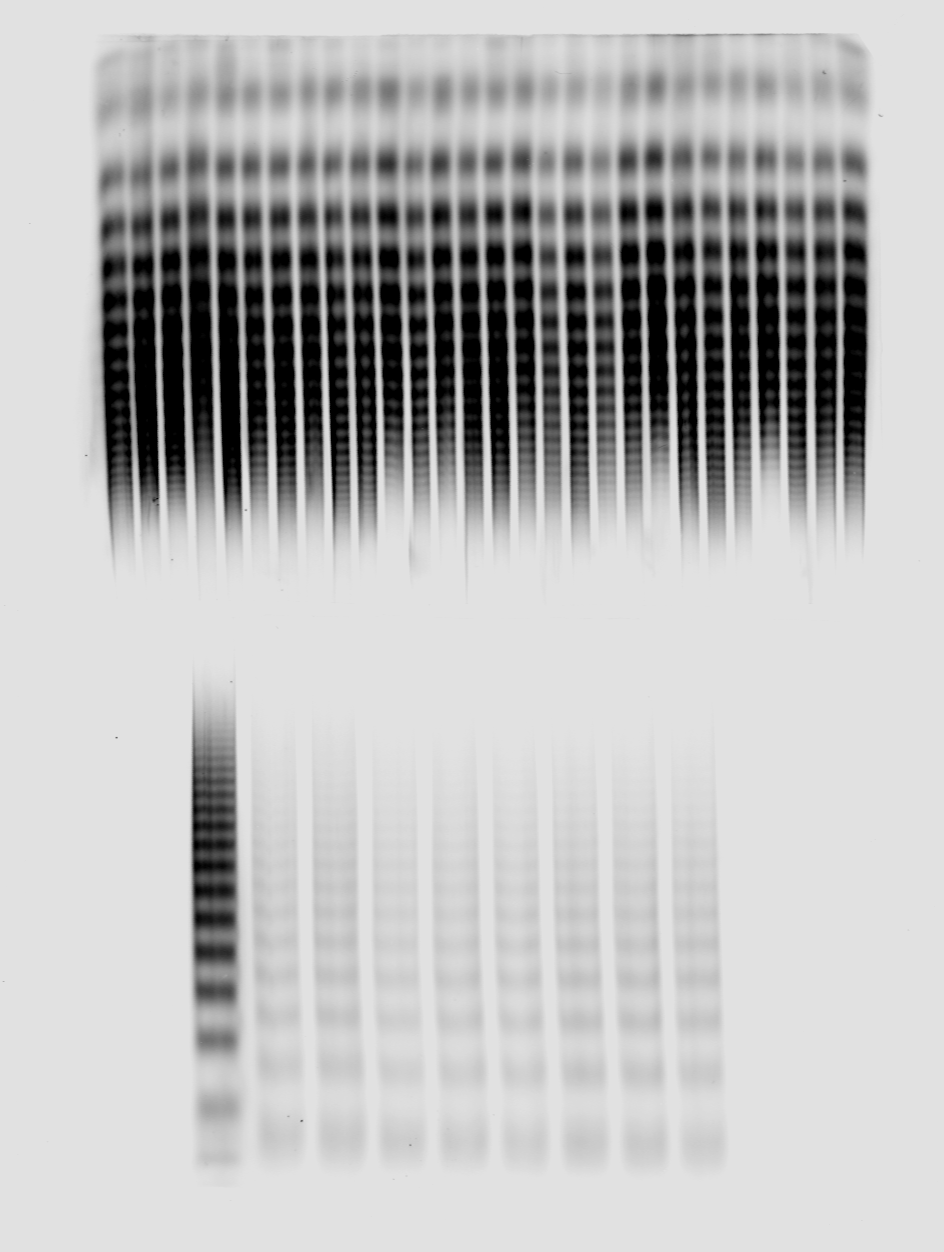

Supplement: Figure 3—figure supplement 1—source data 1. [file elife-71526-fig3-figsupp1-data1.zip › Fig3-fig supp 1B_right.tif]

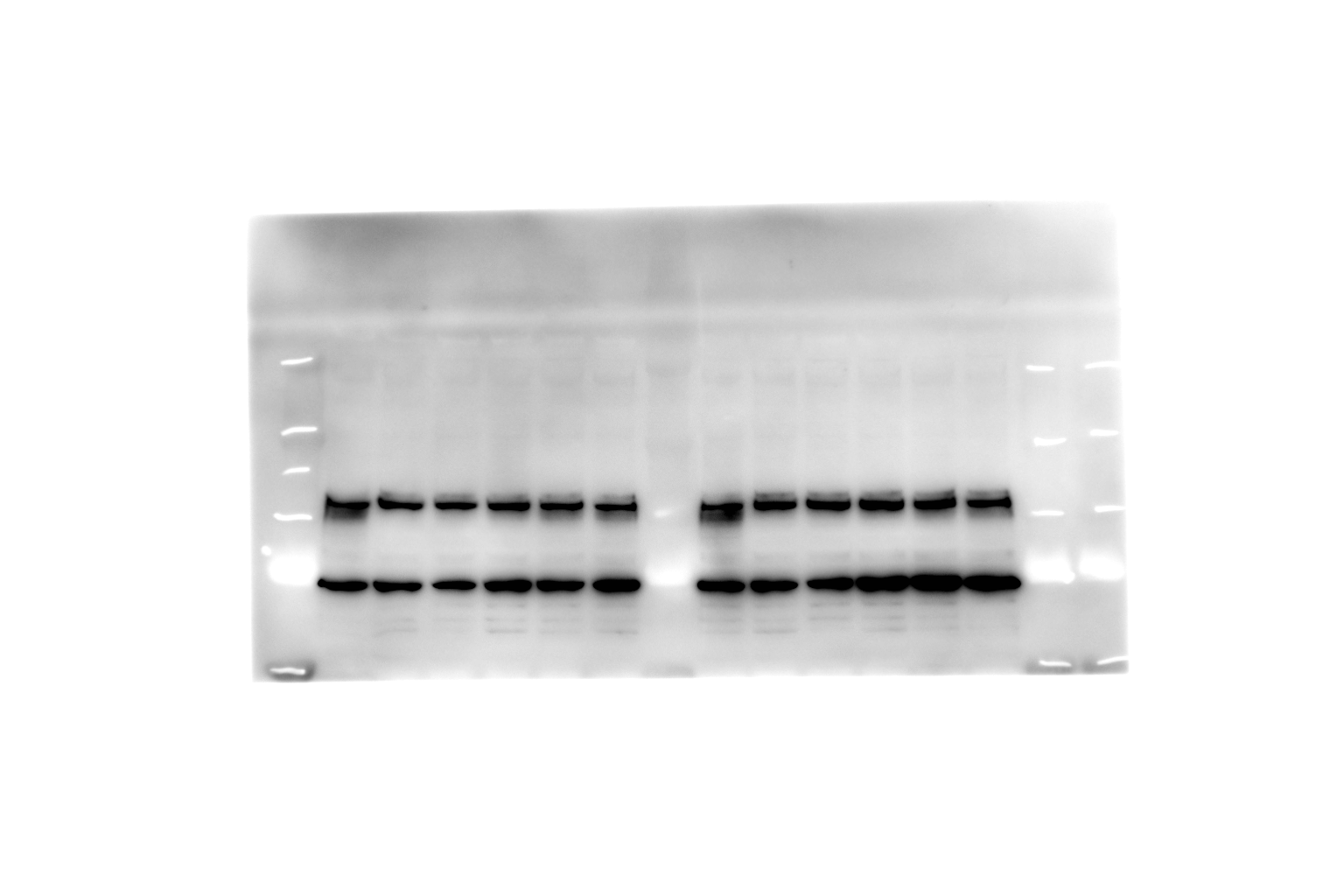

Supplement: Figure 3—figure supplement 1—source data 1. [file elife-71526-fig3-figsupp1-data1.zip › Fig3-fig supp 1A_right.tif]

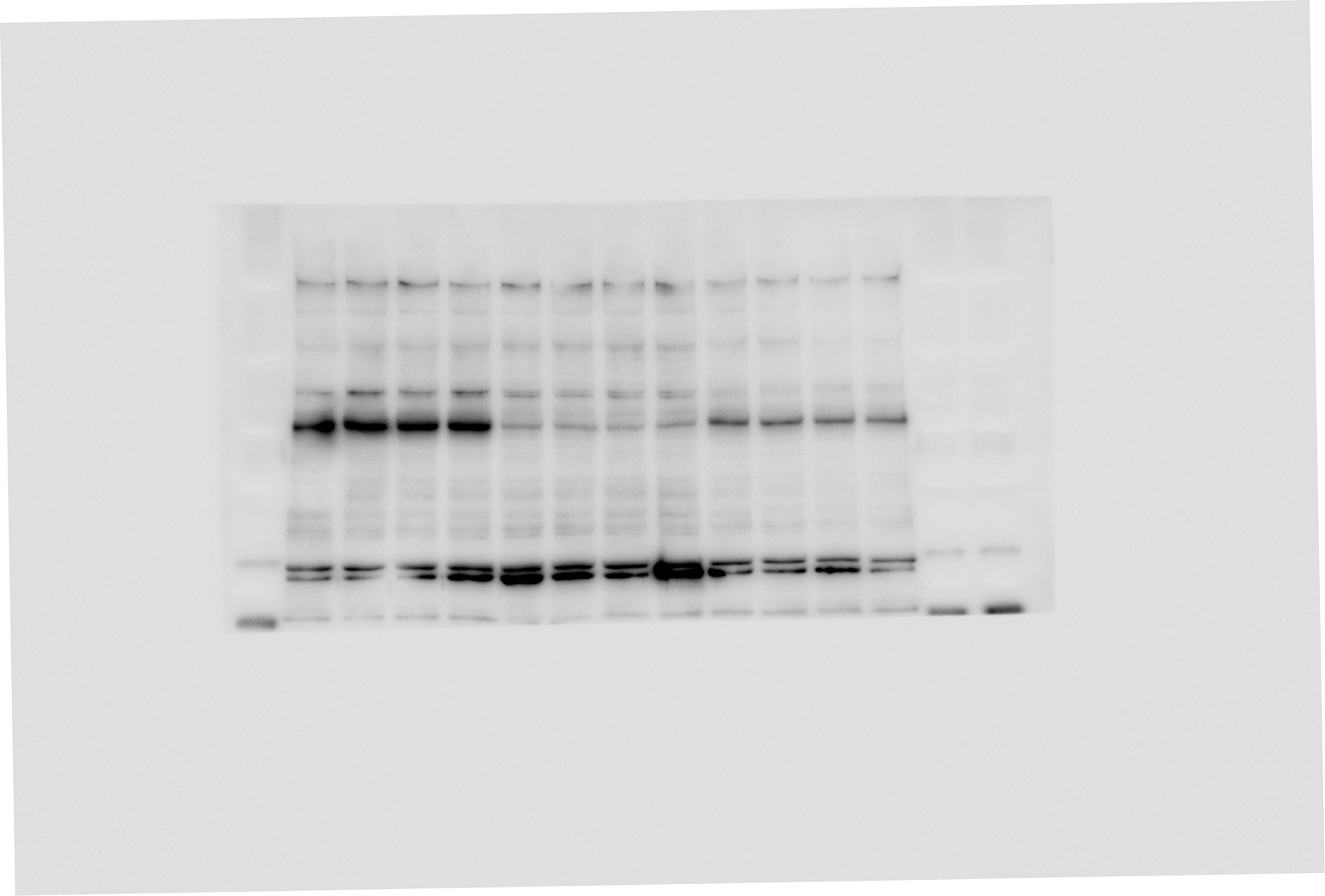

Supplement: Figure 3—figure supplement 1—source data 1. [file elife-71526-fig3-figsupp1-data1.zip › Fig3-fig supp 1A_left.tif]

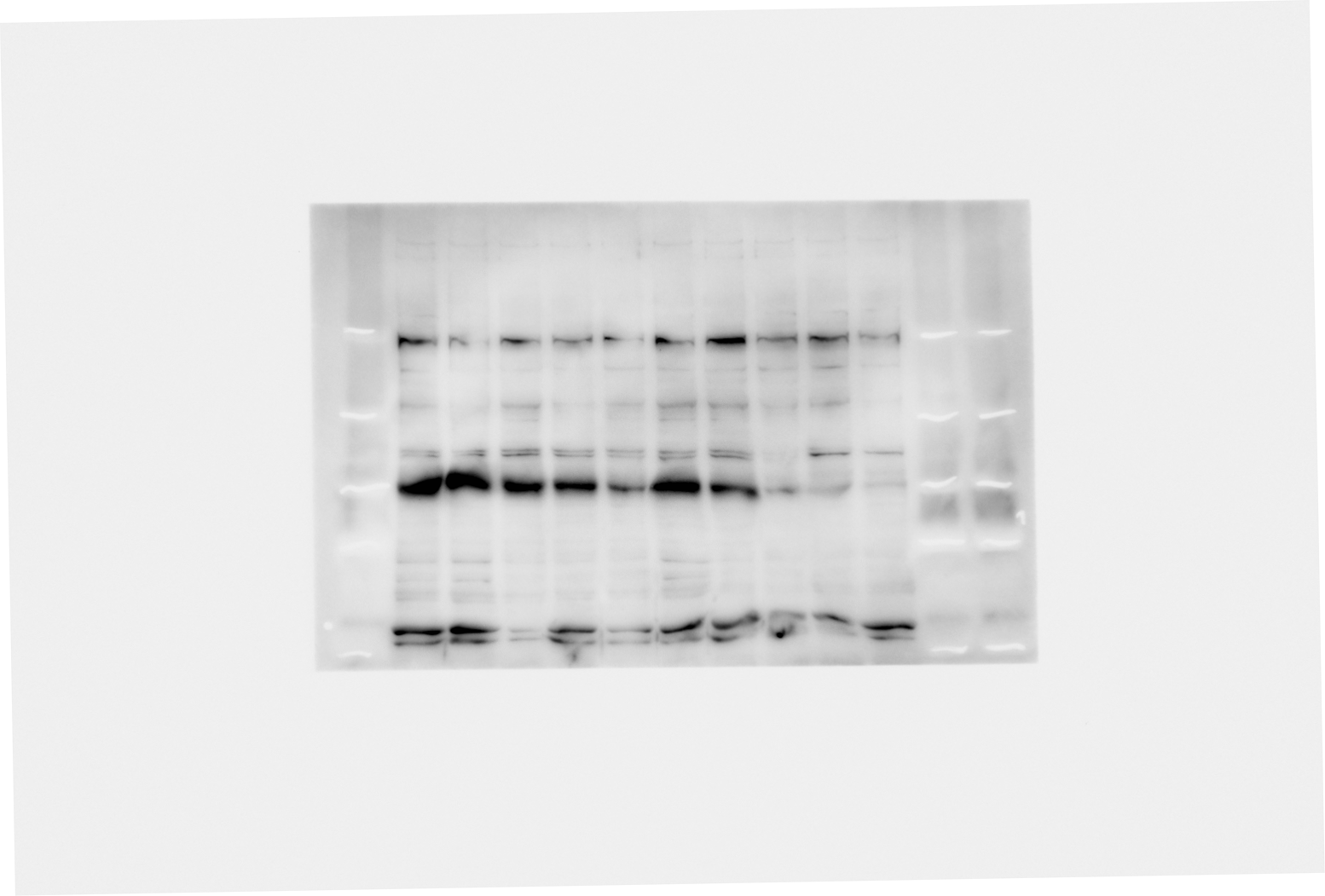

Supplement: Figure 3—figure supplement 1—source data 1. [file elife-71526-fig3-figsupp1-data1.zip › Fig3-fig supp 1C.tif]

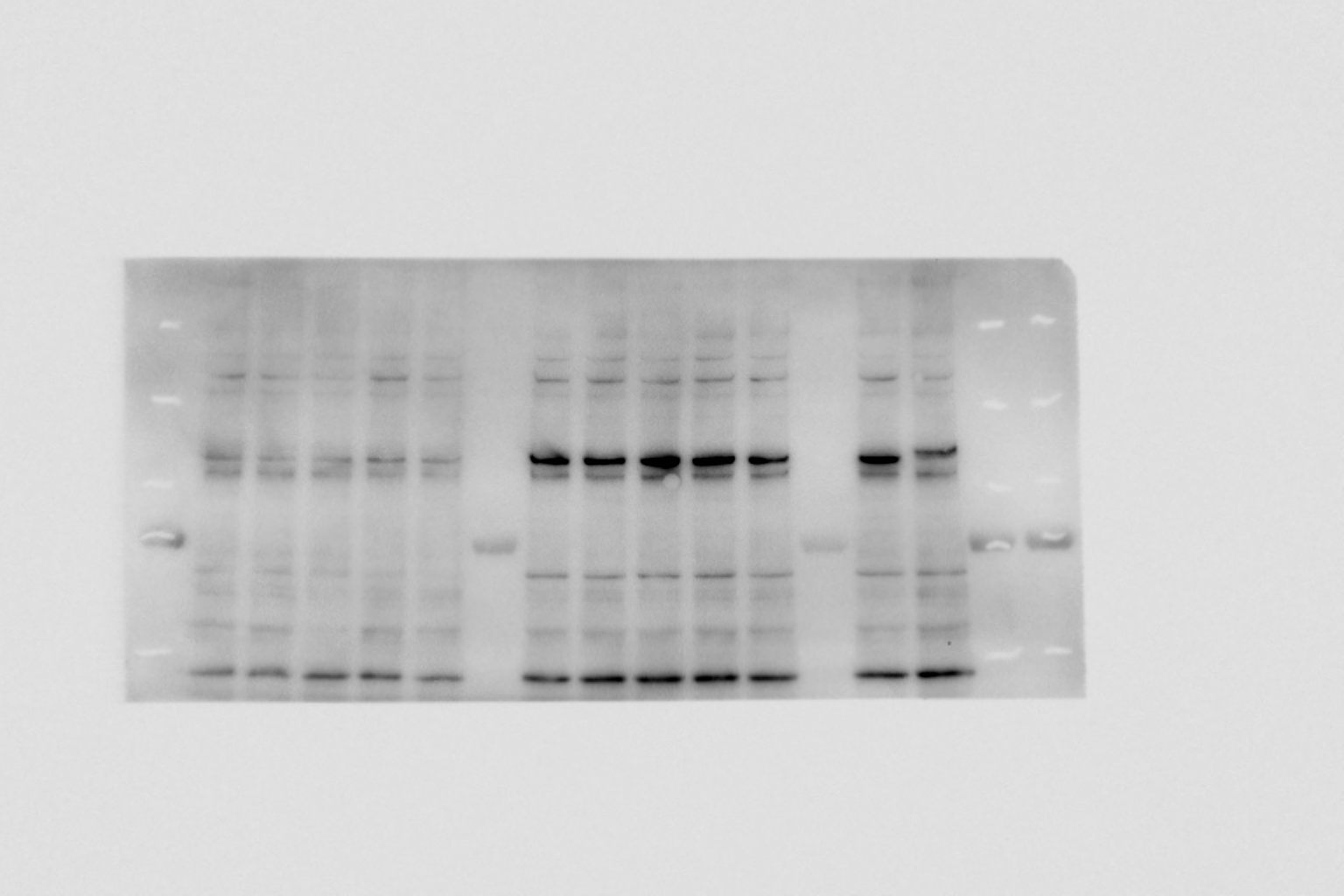

Supplement: Figure 6—figure supplement 2—source data 1. [file elife-71526-fig6-figsupp2-data1.zip › Fig6-fig supp 2A top.tif]

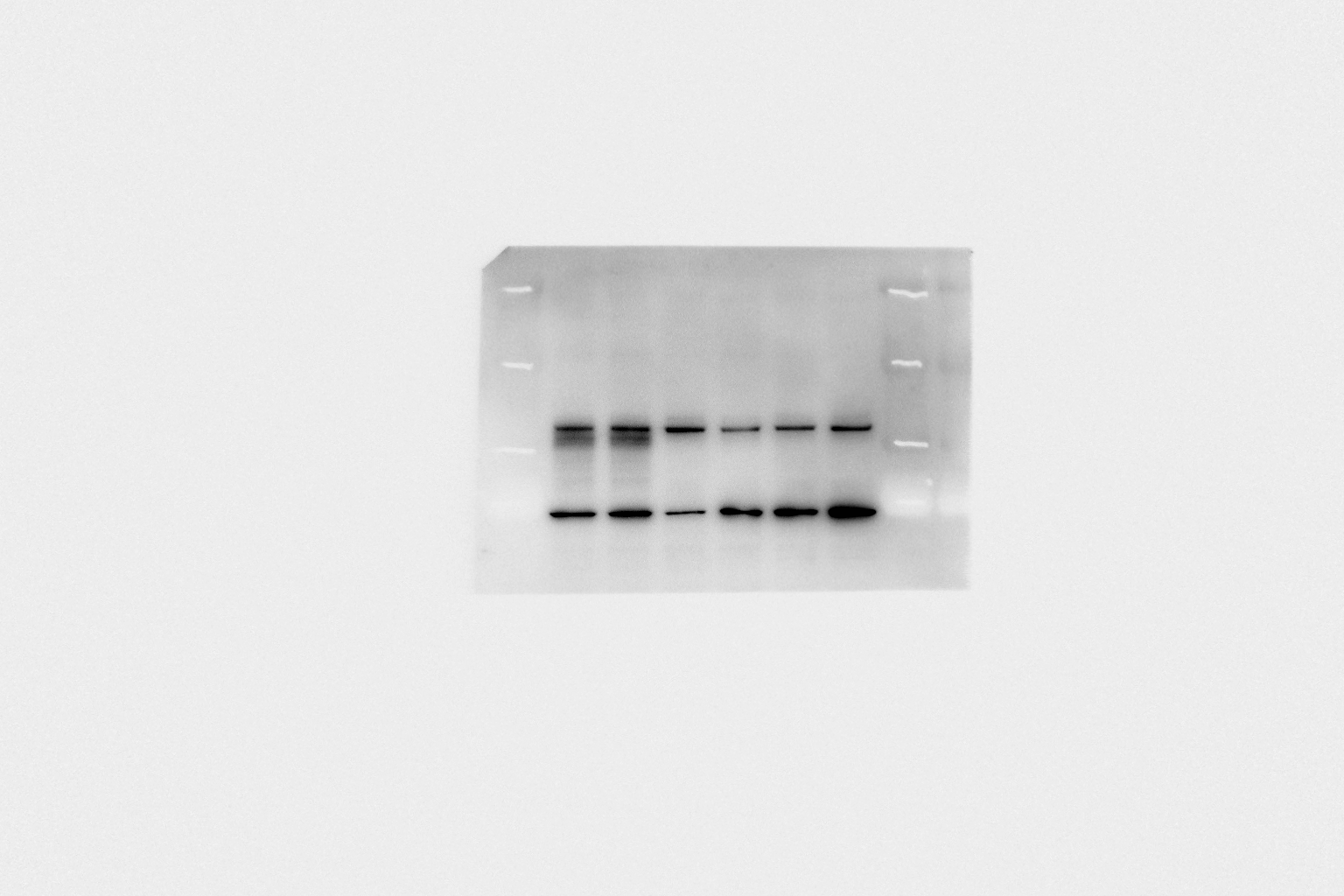

Supplement: Figure 6—figure supplement 2—source data 1. [file elife-71526-fig6-figsupp2-data1.zip › Fig6-fig supp 2B bottom.tif]

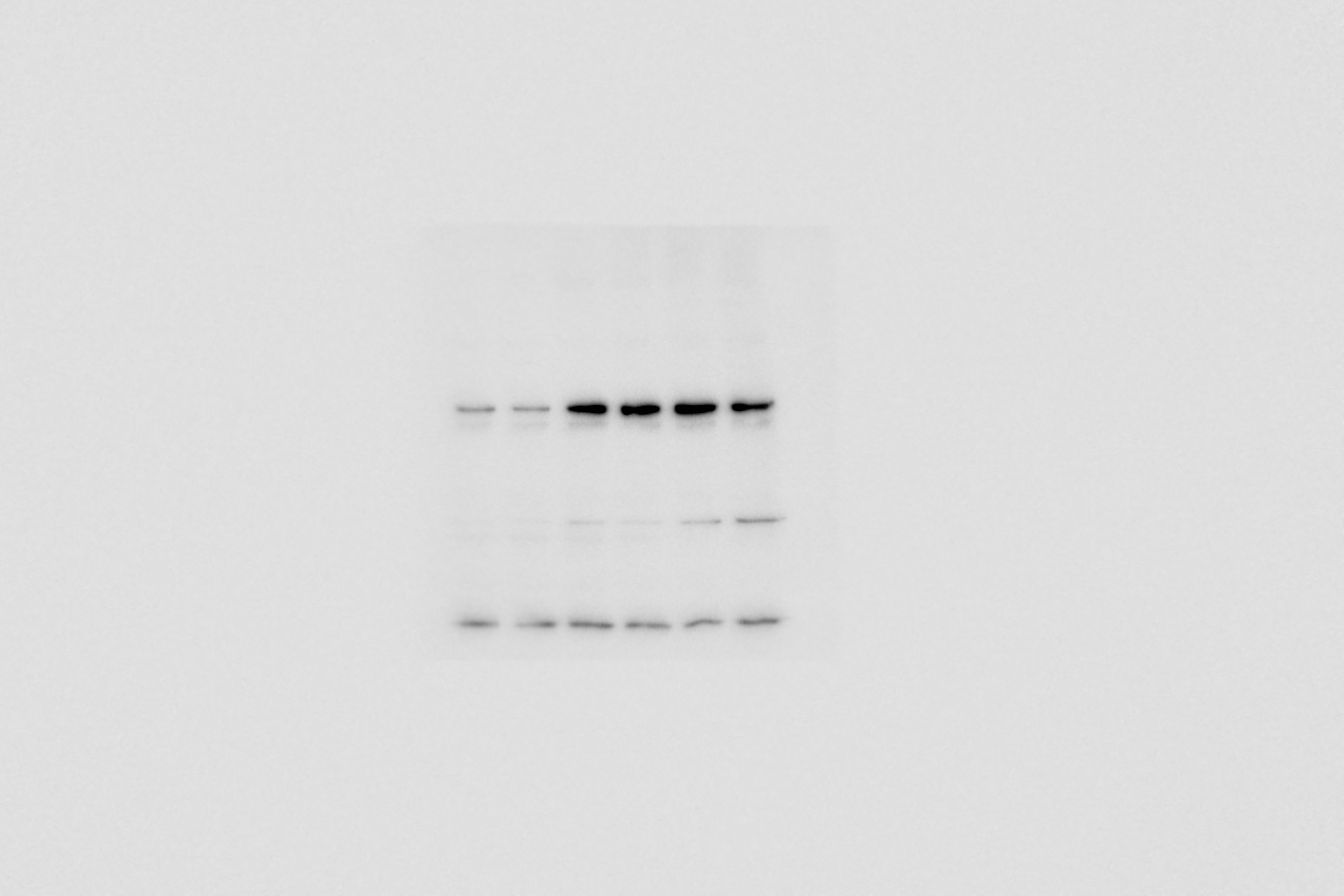

Supplement: Figure 6—figure supplement 2—source data 1. [file elife-71526-fig6-figsupp2-data1.zip › Fig6-fig supp 2B top.tif]

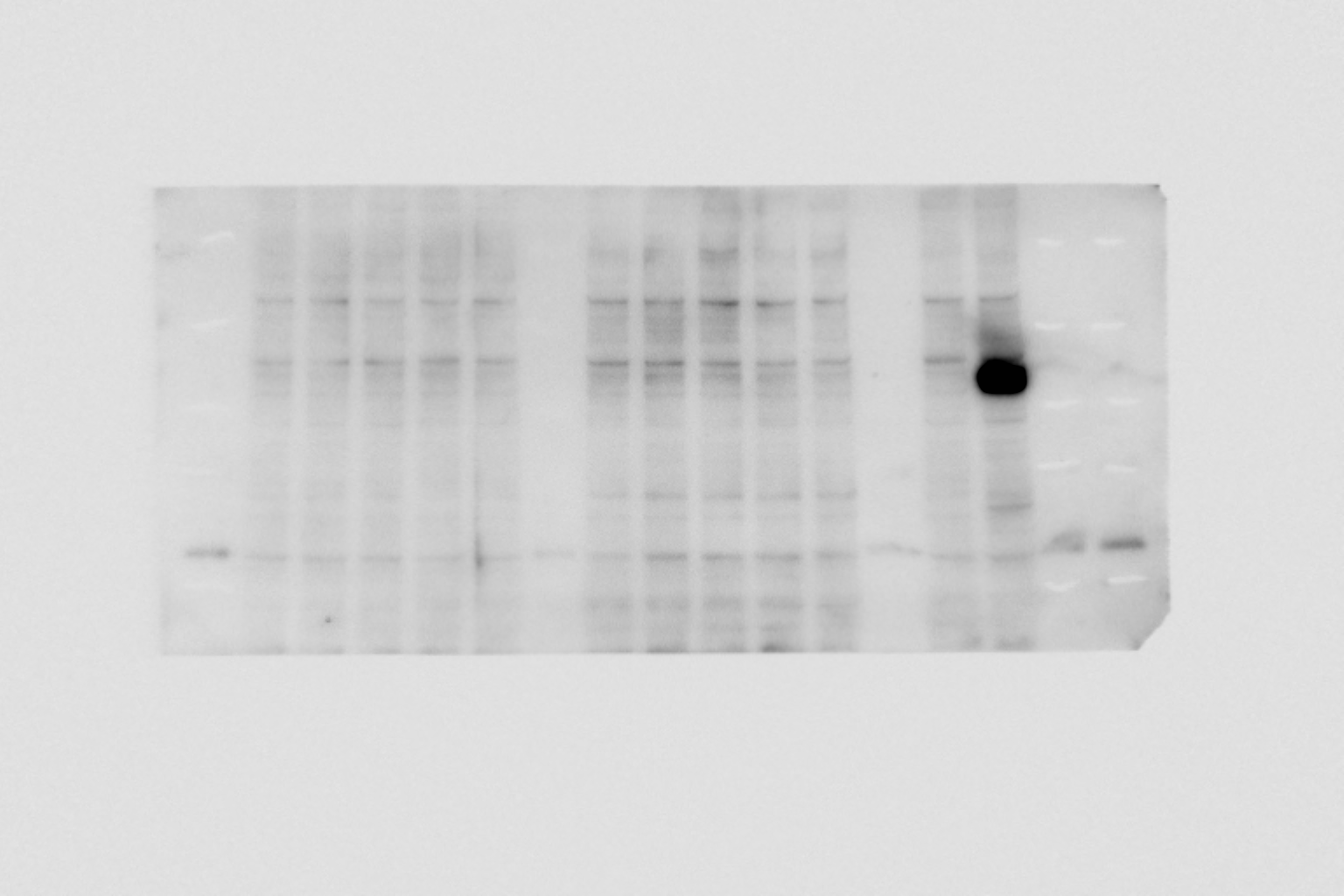

Supplement: Figure 6—figure supplement 2—source data 1. [file elife-71526-fig6-figsupp2-data1.zip › Fig6-fig supp 2A bottom.tif]
