# Supplementary material for: V-ATPase V0a1 promotes Weibel–Palade body biogenesis through the regulation of membrane fission
Source: eLife. 2021 Dec 14;10:e71526. doi: 10.7554/eLife.71526 (PMC8718113; doi:10.7554/eLife.71526)
Supplement: Figure 2—figure supplement 1—source data 1. [file elife-71526-fig2-figsupp1-data1.zip › Fig 2-fig supp 1_labeled.pptx]

## Slide 1
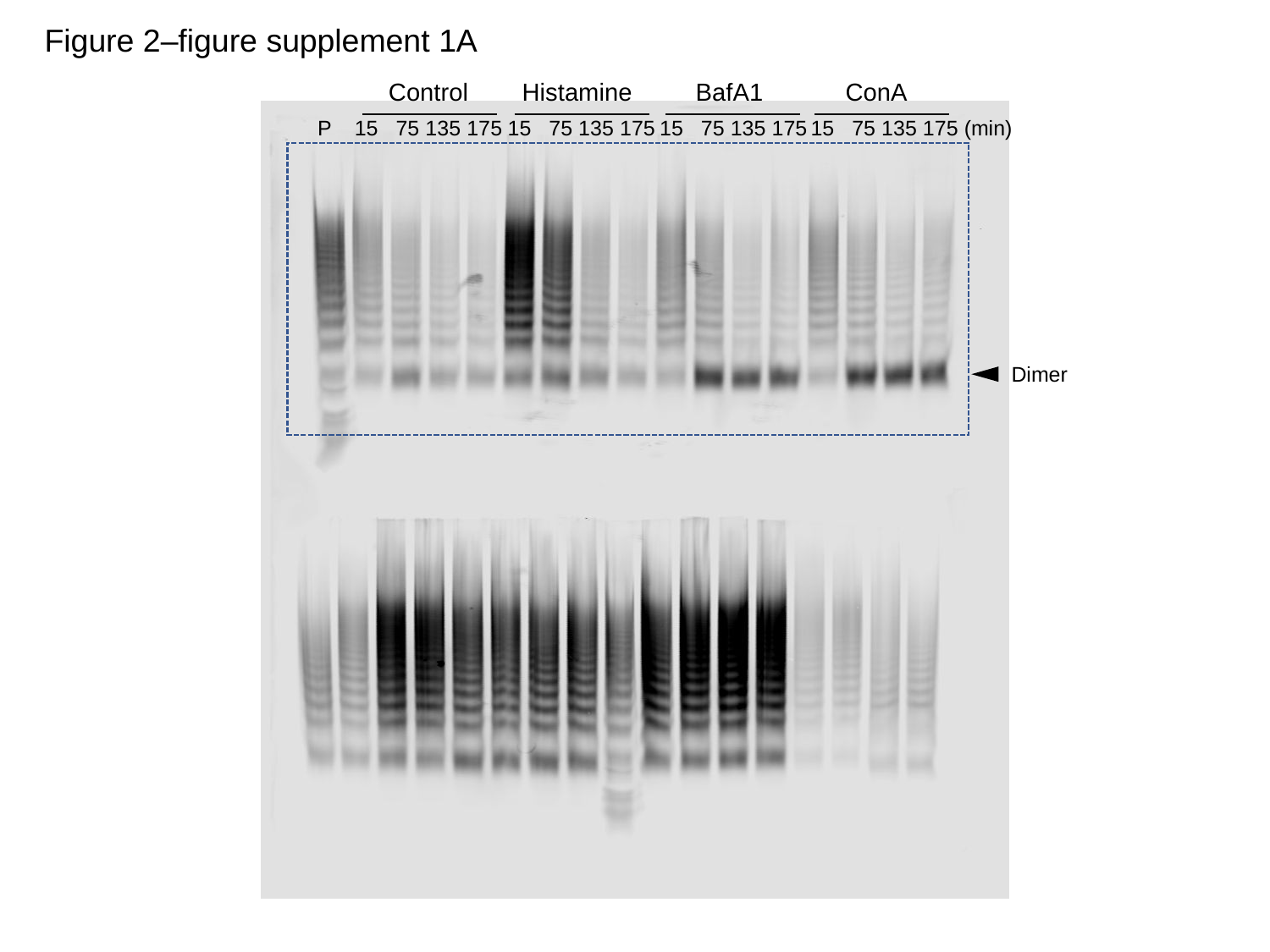

Figure 2–figure supplement 1A
Control
Histamine
BafA1
ConA
P
15 75 135 175
15 75 135 175
15 75 135 175
15 75 135 175 (min)
Dimer

## Slide 2
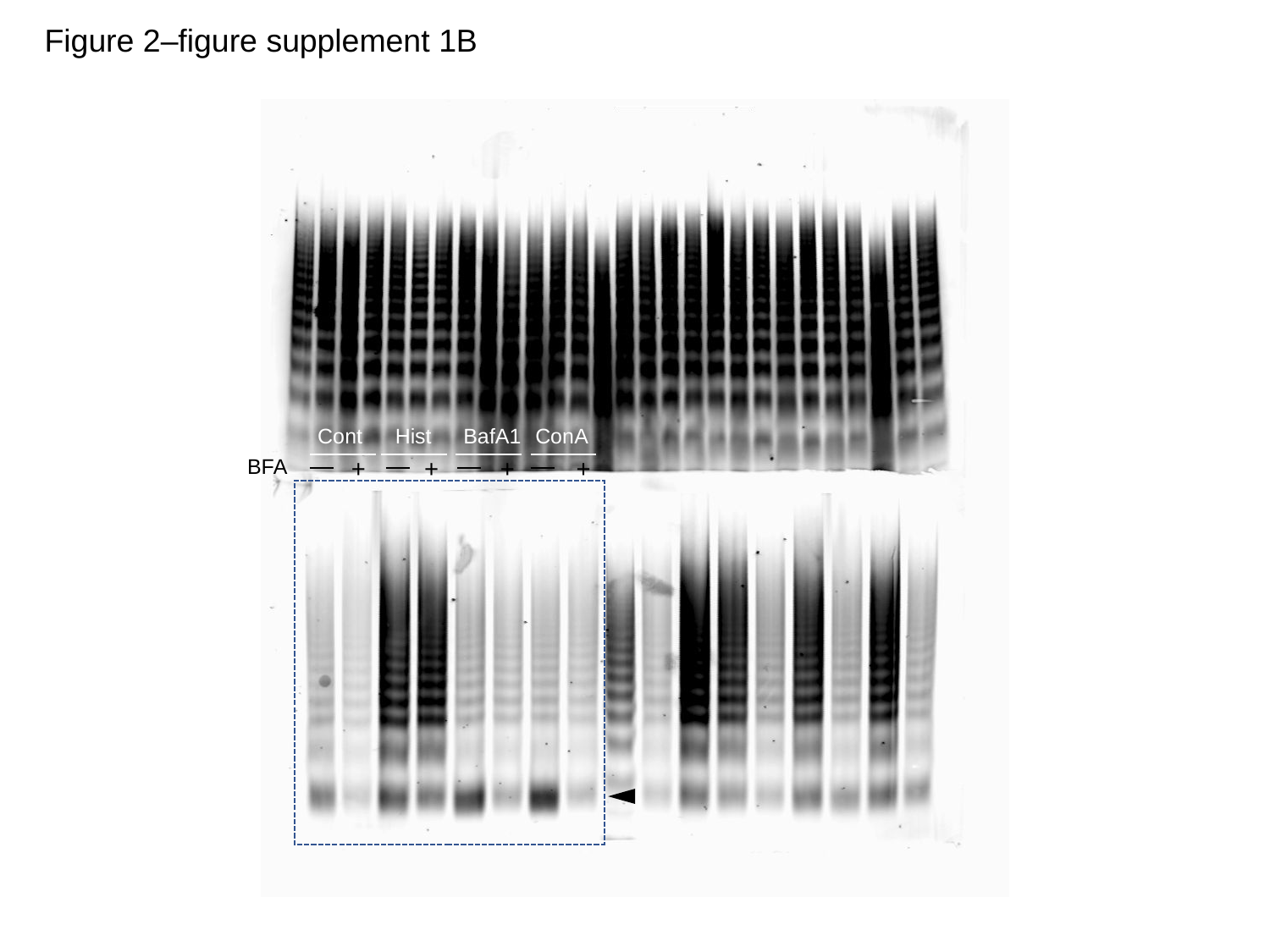

Figure 2–figure supplement 1B
Cont
Hist
BafA1
ConA
BFA
+
+
+
+
