# Supplementary material for: V-ATPase V0a1 promotes Weibel–Palade body biogenesis through the regulation of membrane fission
Source: eLife. 2021 Dec 14;10:e71526. doi: 10.7554/eLife.71526 (PMC8718113; doi:10.7554/eLife.71526)
Supplement: Figure 3—figure supplement 1—source data 1. [file elife-71526-fig3-figsupp1-data1.zip › Fig 3-fig supp 1_labeled.pptx]

## Slide 1
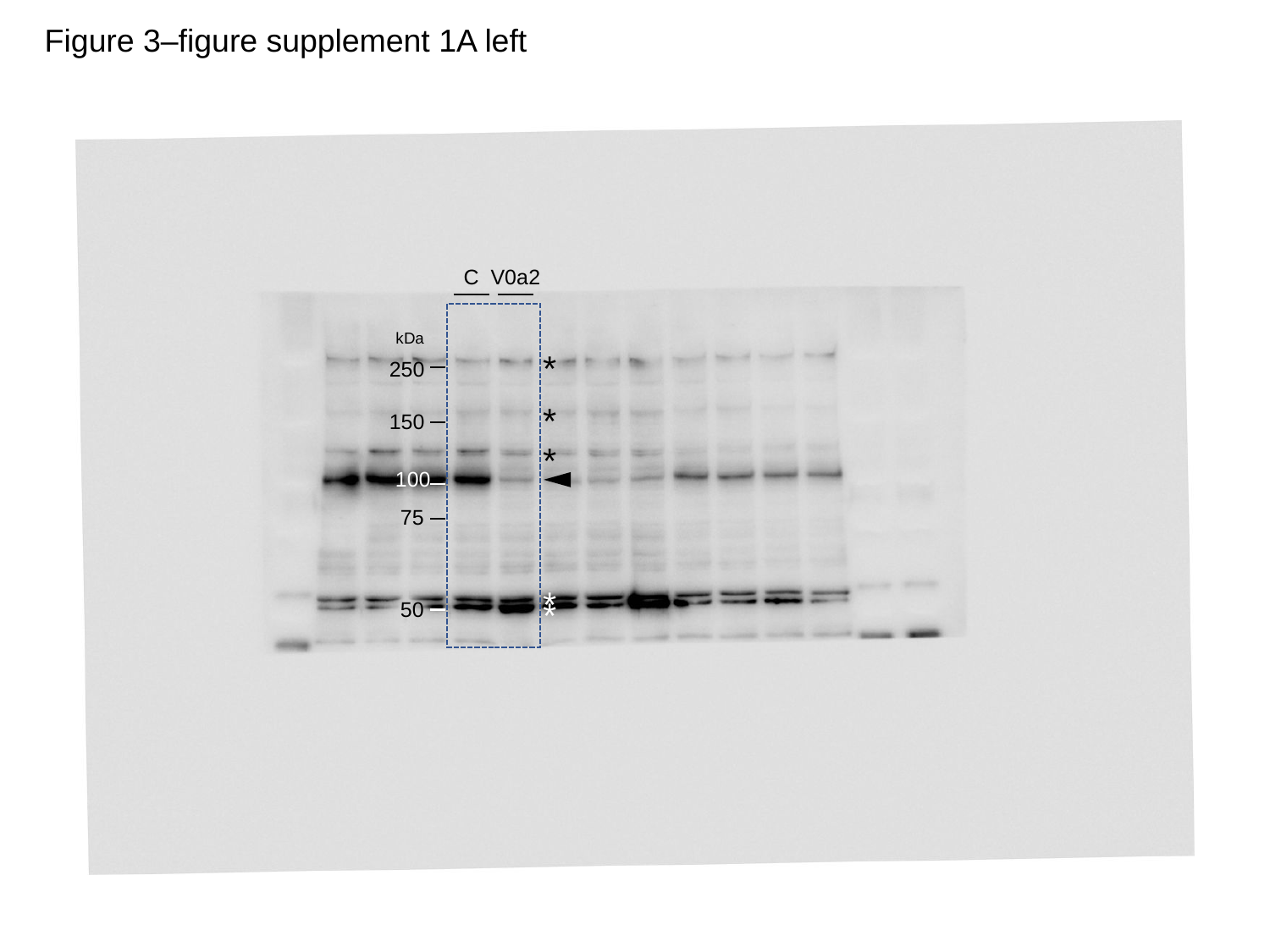

Figure 3–figure supplement 1A left
C
V0a2
kDa
*
250
*
150
*
100
75
*
*
50

## Slide 2
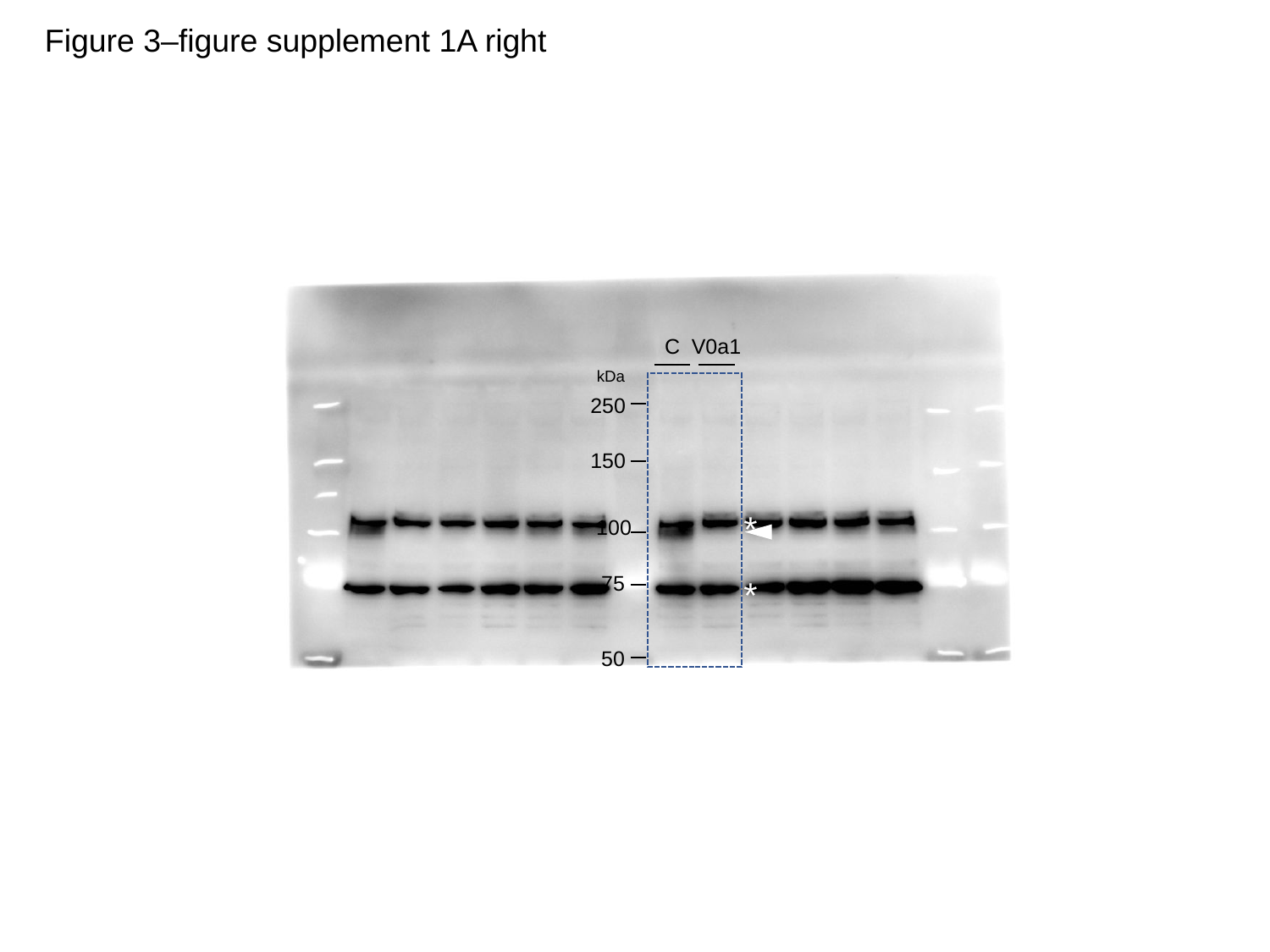

Figure 3–figure supplement 1A right
C
V0a1
kDa
250
150
*
100
75
*
50

## Slide 3
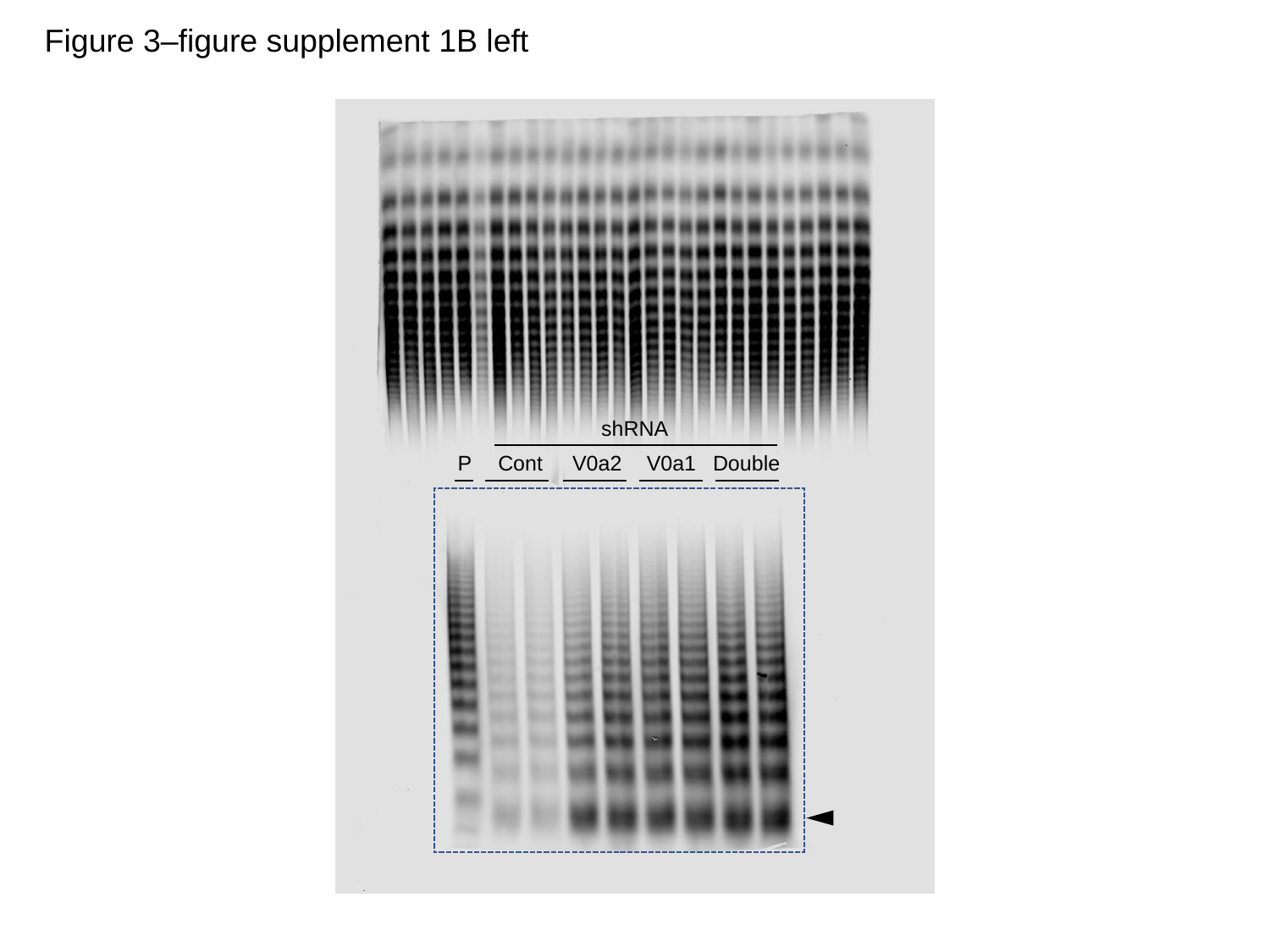

Figure 3–figure supplement 1B left
shRNA
P
Cont
V0a2
V0a1
Double

## Slide 4
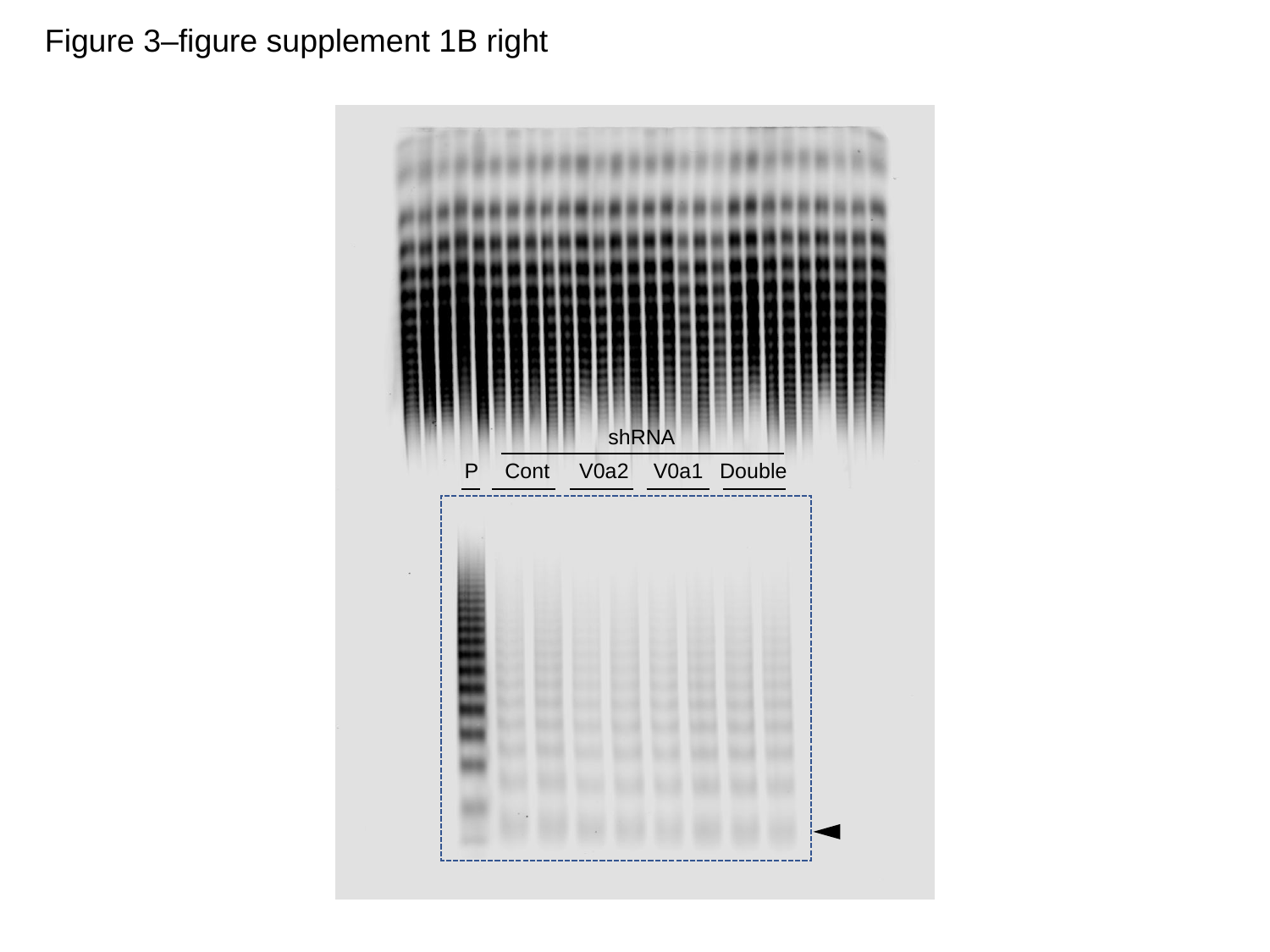

Figure 3–figure supplement 1B right
shRNA
P
Cont
V0a2
V0a1
Double

## Slide 5
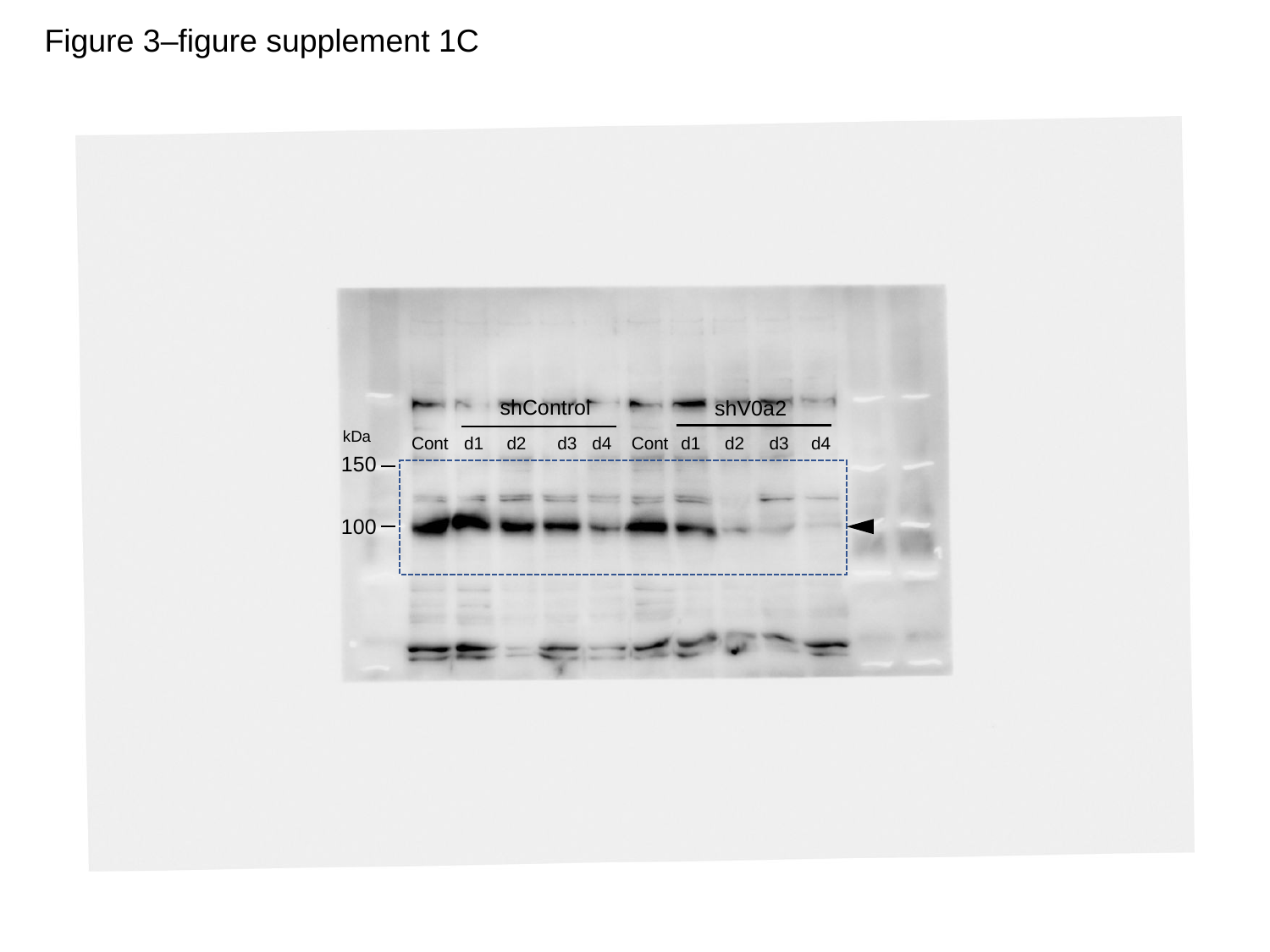

Figure 3–figure supplement 1C
shControl
shV0a2
kDa
Cont
d1
d2
d3
d4
Cont
d1
d2
d3
d4
150
100
