# Supplementary material for: V-ATPase V0a1 promotes Weibel–Palade body biogenesis through the regulation of membrane fission
Source: eLife. 2021 Dec 14;10:e71526. doi: 10.7554/eLife.71526 (PMC8718113; doi:10.7554/eLife.71526)
Supplement: Figure 6—figure supplement 2—source data 1. [file elife-71526-fig6-figsupp2-data1.zip › Fig 6-fig supp 2_labeled.pptx]

## Slide 1
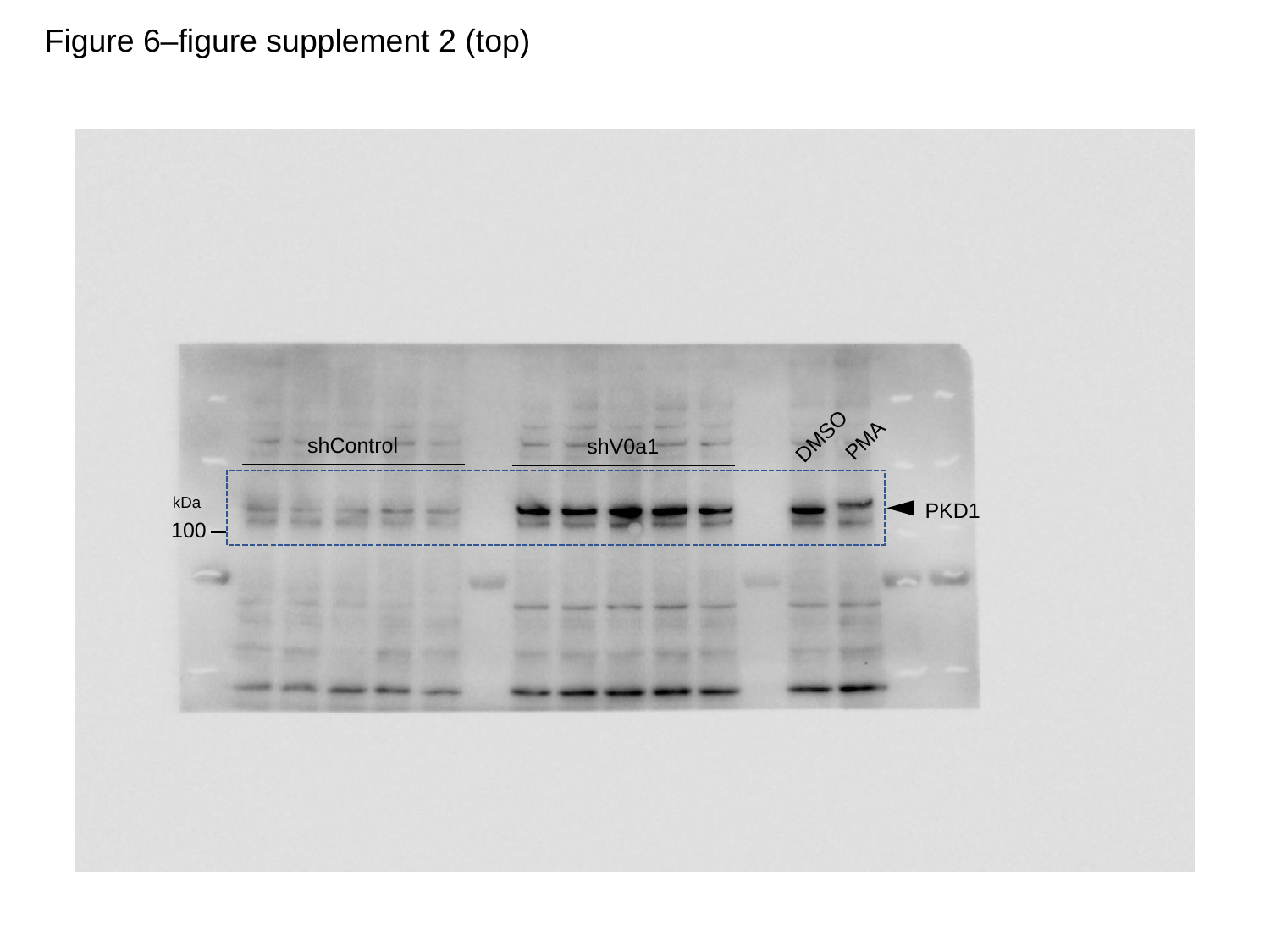

Figure 6–figure supplement 2 (top)
PMA
DMSO
shControl
shV0a1
kDa
PKD1
100

## Slide 2
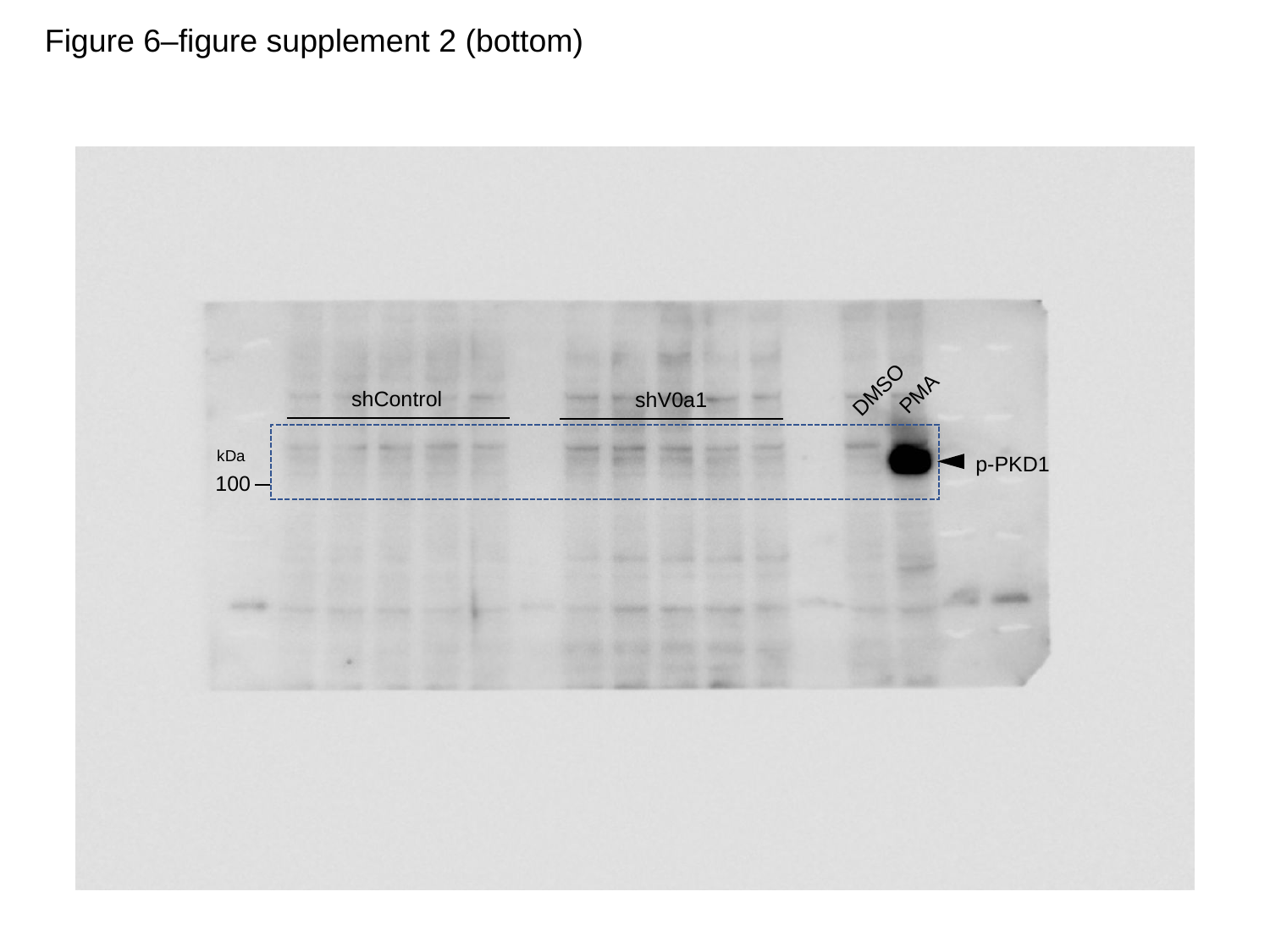

Figure 6–figure supplement 2 (bottom)
PMA
DMSO
shControl
shV0a1
kDa
p-PKD1
100

## Slide 3
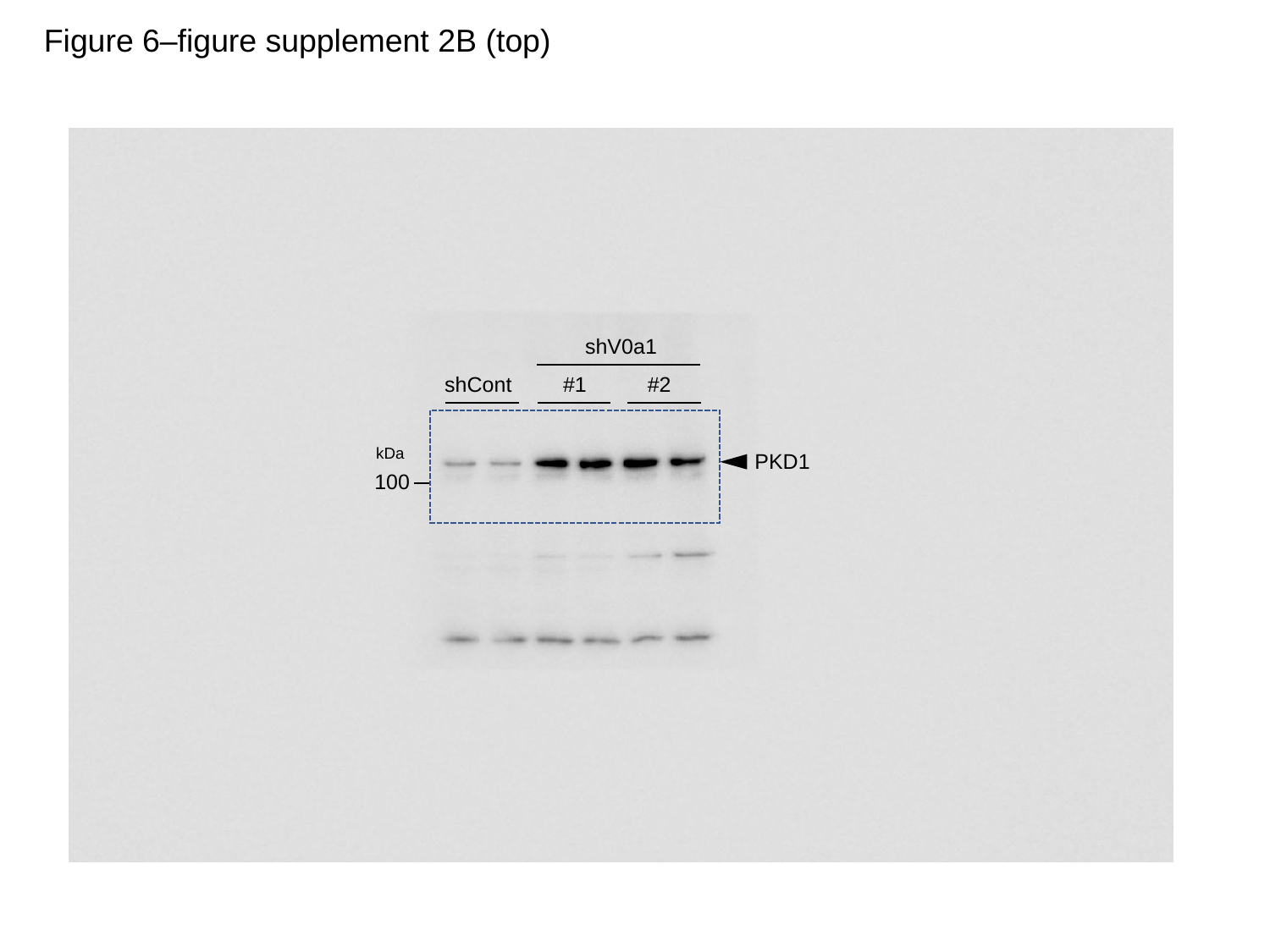

Figure 6–figure supplement 2B (top)
shV0a1
shCont
#2
#1
kDa
PKD1
100

## Slide 4
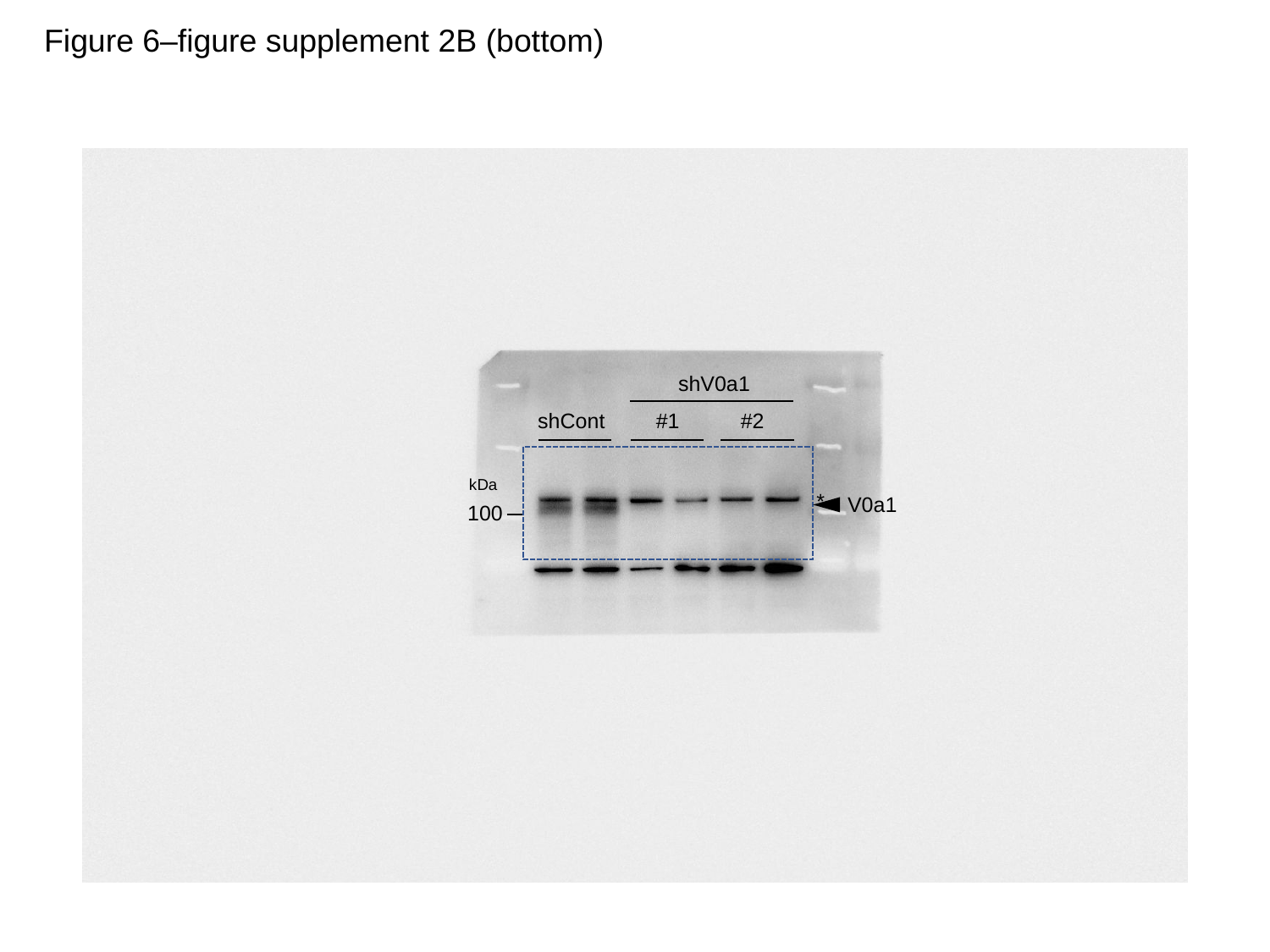

Figure 6–figure supplement 2B (bottom)
shV0a1
shCont
#2
#1
kDa
*
V0a1
100
